# Clinicians’ views on working with anorexia nervosa and autism spectrum disorder comorbidity: a qualitative study

**DOI:** 10.1186/s12888-017-1455-3

**Published:** 2017-08-10

**Authors:** Emma Kinnaird, Caroline Norton, Kate Tchanturia

**Affiliations:** 1 King’s College London, London, Institute of Psychiatry, Psychology and Neuroscience, Department of Psychological Medicine, London, UK; 2 Eating Disorders National Service, South London and Maudsley NHS Foundation Trust, London, UK; 3 Illia State University, Tbilisi, Georgia; 4 PO59, King’s College London, IoPPN, Psychological Medicine, 16 De Crespigny Park, London, SE5 8AF UK

**Keywords:** Anorexia nervosa, Autism spectrum disorder, Treatment, Qualitative study, Comorbidity

## Abstract

**Background:**

Anorexia nervosa (AN) and autism spectrum disorder (ASD) form a relatively common comorbidity, with poorer illness outcomes and poorer responses to treatments for AN compared to individuals without ASD. However, the treatment of this comorbidity remains poorly understood: no research to date has examined how clinicians currently approach treating AN/ASD. This study aimed to explore the experiences of clinicians working with comorbid AN/ASD using qualitative methods in order to identify areas for future improvement.

**Methods:**

Interviews with individual clinicians (*n* = 9) were carried out and explored using thematic analysis.

**Results:**

The findings suggest that many clinicians lack confidence in treating this comorbidity, which requires specific changes to treatment to accommodate the issues raised by comorbid ASD. At present, any adaptations to treatment are based on the previous experience of individual clinicians, rather than representing a systematic approach.

**Conclusions:**

Further research is needed to empirically assess potential treatment modifications for this group and to establish guidelines for best clinical practice.

**Electronic supplementary material:**

The online version of this article (doi:10.1186/s12888-017-1455-3) contains supplementary material, which is available to authorized users.

## Background

There has been an upsurge in research over the past few years documenting the relationship between autistic spectrum disorder (ASD) and anorexia nervosa (AN), first identified by Gillberg in 1983 [1]. ASD is a neurodevelopmental disorder characterised by problems in social and communicative functioning and restricted patterns of behaviour with onset in the early developmental period [2]. By contrast, AN is a severe eating disorder, characterised by a low body weight due to restricted energy intake, a fear of gaining weight, and an undue influence of shape and weight on their self-evaluation [2]. Unlike ASD, AN does not typically develop until adolescence or early adulthood [3].

Despite these apparent differences, it has been consistently demonstrated across a number of studies that ASD traits are elevated in some individuals with AN, and that these traits may contribute towards some of the psychopathological features associated with AN [4–7]. Although research in this area is ongoing, it appears that there is a higher prevalence of diagnosed ASD in individuals with AN compared to the healthy population [8–10]. This relationship between the two conditions appears to be underpinned by common underlying neuropsychological and social problems, with individuals with AN exhibiting neurocognitive problems more typically associated with ASD. AN has been associated with significant inefficiencies in theory of mind, cognitive flexibility and central coherence [11–16]. Individuals with AN are also characterised by poor social and emotional functioning [17, 18], including interpersonal problems [19], impaired facial emotion recognition [20, 21], diminished facial emotion expression [22, 23], and social anhedonia [24, 25]. These problems are associated with a longer illness duration, higher illness severity, and poor treatment outcomes [13, 24–27]. Consequently, Treasure & Schmidt (2013) have proposed that these cognitive, socio-emotional and interpersonal problems in fact act as maintaining factors for the disorder itself [28]. It is therefore possible that the presence of underlying ASD traits effectively maintains AN [5, 29].

A key challenge in examining the relationship between AN and ASD is the fact that the starvation, depression and anxiety symptoms present in AN can also contribute to these cognitive and social problems, rather than indicating the presence of an underlying neurodevelopmental disorder [30–32]. However, these difficulties have been found to precede the onset of AN in childhood, and to persist to a less severe extent following weight gain and recovery [13, 18, 27, 31, 33–35]. This suggests that these problems may for some individuals represent underlying traits that preceded the onset of the disorder, rather than symptoms resulting from starvation: a recent case series study identified a number of individuals with AN and ASD traits with longstanding neurodevelopmental difficulties, confirming a genuine overlap in some individuals between the two disorders [9].

Nonetheless, this overlap between potential symptoms of starvation and the possibility of comorbid ASD can make diagnosis difficult if the patient is still in the acute stage of their illness: a longitudinal cohort study on ASD and AN comorbidity found that a number of patients with AN had an ASD diagnosis at one stage of the study, but were then later found to no longer meet the criteria [36]. Consequently, not all psychopathological features seen in individuals with AN that resemble ASD are associated with an underlying neurodevelopmental disorder.

Although only a limited number of patients with AN show ASD traits, understanding the relationship between ASD and AN is crucial because this comorbidity has been related to poorer illness outcomes [29]. Individuals with high ASD traits have been found to respond more poorly to psychological treatments [37], with a clinical case study of two children with comorbid AN and ASD suggesting that the rigidity and low introspection characteristic of ASD hindered responses to traditional therapeutic programmes [38]. Consequently, it has been suggested that individuals with comorbid AN and ASD require adapted or targeted treatment programmes [31, 38]. Treatments such as cognitive behavioural therapy (CBT) have previously been adapted for individuals with ASD and comorbid anxiety or depression [39]. However, there has been no research to date on how clinicians currently approach treating comorbid AN and ASD, and how more typical therapeutic approaches may be adapted for these patients.

This paper presents an exploratory qualitative study of the experiences of clinicians treating comorbid AN and ASD. The aim is to understand how clinicians approach treating comorbid AN and ASD, and how they adapt their typical therapeutic techniques for these patients. This paper will focus on the following research questions:

1. How do clinicians react if their patient presents with AN and comorbid ASD or ASD traits?
2. What specific issues do clinicians face when treating comorbid ASD and AN?
3. What treatment techniques or approaches are effective in treating comorbid ASD and AN?

## Methods

### Design

The study adopted a qualitative design, using semi-structured interviews to explore therapist experiences of treating people with comorbid AN and ASD. The study was part of a wider clinical service improvement project approved by South London and Maudsley NHS trust governance committee.

### Participants

Participants were recruited from the South London and Maudsley NHS Foundation Trust Eating Disorders Service outpatient and daycare departments. Participants were all currently working with the outpatient team treating adults with EDs. Participants were invited prior to data collection through email due to having a minimum of 3 years of clinical experience working with patients with ED. All participants had a prior professional relationship with the interviewer. Ten clinicians were invited to take part, and nine agreed to take part and so formed the final cohort. One clinician did not feel comfortable discussing the interview topic and so was not included in the sample. This study included a range of clinical backgrounds, including nurse therapists, cognitive behavioural therapists, a cognitive analytical therapist, a psychotherapist, a dietician, an occupational therapist. All participants were female.

### Data collection

Written informed consent was acquired prior to the interview. The interviews were semi-structured with open ended questions, conducted by CN and recorded by EK. No other individuals were present. CN is a female team leader of the outpatient ED team, with a background in nursing and EDs. Participants were asked one general question: “What do you do if your patient with an eating disorder has an autism spectrum disorder diagnosis or traits?” (Additional file 1). The interviewer then asked follow-up questions about specific themes that emerged in each interview. The average length of each interview was 20 min. No repeat interviews were conducted.

All interviews took place within the outpatient department as the therapist’s place of work. Participants were informed of the study’s purpose prior to data collection, and made aware that the study was being conducted due to author interest in improving service provision for individuals with ASD and AN. Interviews were audio recorded and transcribed, with any identifying information removed at the point of transcription. EK additionally made field notes during data collection. Transcript were not returned to participants. Following the nine interviews, it was judged that a point of data saturation had been reached and recruitment ceased.

### Data analysis

Data were analysed using a thematic analysis methodology, which aims to identify and summarise relevant patterns in the data [40]. Data were read and re-read in order to achieve familiarity with the data by all authors. Data were then interpreted and coded line by line by the first author (EK) using NVivo 11, deriving themes from the data relevant to the research question. The coding frame at this stage included a focus on therapist perceptions of having a patient with AN/ASD, difficulties treating AN/ASD and therapeutic techniques used. These codes were reviewed and grouped into key themes, summarised in the Results section. All authors met to achieve consensus on the themes. Participants did not give feedback on the findings.

## Results

Themes and sub themes identified in the analysis are summarised in Table 1. The number of participants endorsing each item is given to highlight key patterns and ensure the transparency of analysis. Themes with low participant endorsement but high relevance to the research question were retained in the analysis.

**Table 1:** Summary of themes and sub-themes

Theme	Sub-Theme	Participant endorsement
Therapist reactions to having a patient with ASD	Lack of confidence	1, 2, 3, 4, 6, 7
	Information seeking	2, 4, 5, 7
	Diagnostic referral	2, 4, 5, 8
Specific issues in treating comorbid ASD and AN	Difficulty treating	1, 2, 4
	Communication problems	1, 2, 4, 5, 6, 9
	Emotions	1, 5, 8
	AN/ASD overlap	1, 2, 3, 7, 8
	Sensory	5
Techniques used to treat comorbid ASD/AN	Patient-driven process	1, 2, 4, 7, 8
	Adaptations to therapy	1, 5, 9
	Adaptations to communicative style	2, 6, 9
	Specific modifications	1, 4, 5, 8, 9

### Clinician reflections on having a patient with ASD

A common theme throughout the interviews was a lack of clinician confidence or experience in treating patients with comorbid ASD (60% of participants). Consequently, when treating a patient already diagnosed with ASD, clinicians suggested that their first reaction would be to initially look for more information on the subject. As well as looking for resources online or reading books, clinicians described seeking help from other, more experienced/senior members of the treatment team:

“So, but I don’t know a huge amount about ASD so I’d certainly find, speak to somebody that knows a bit more about it or look up something just to think about what I might need to be aware of.” – Participant 7.

Where the patient was exhibiting ASD traits but had no prior diagnosis, a number of clinicians suggested that they would refer the individual to a specialist ASD service for the assessment. However, there did not appear to be clear or common pathways for assessment referrals, with participants variously suggesting that they would consult a specialist registrar doctor (Participant 4), the team lead clinical psychologist (Participant 4), specialist services (Participant 5) or carrying out a brief assessment themselves (Participant 5).

Participant 5 also emphasised that she would not do this automatically, but would rather consider a referral on an individual basis:

“If we’re thinking about whether someone who’s undiagnosed who may have an ASD diagnosis, I will be- along the course of therapy- thinking about whether it’s going to be helpful to pursue that or not. Of course, with some people it’s not helpful to even think about diagnosis - it’s not going to be something that they’re going to gain from it.”

Participant 8 also noted that she would wait until the patient was weight restored until she referred for a diagnosis, “because low weight can actually make it seem like they have ASD or ASD traits”.

### Specific issues in treating comorbid ASD and AN

Clinicians raised a number of issues they had previously encountered when treating patients with comorbid ASD and AN, with Participant 2 noting “it was difficult to work with”. A common difference noted by clinicians compared to non-ASD patients was their communication styles:

“You listen to the way they respond, if you like, and sometimes their responses are very short compared to somebody that doesn’t have ASD. You know sometimes, you start somebody off on something and they can roll on for ages telling you about a situation. But I find with autism every time you ask for something you get a sentence back - you don’t get, sometimes it’s quite curt, you know, almost to the point of rude but not rude because that’s how they respond” – Participant 1.

Participant 1 suggested that these communication problems made building a therapeutic relationship more difficult- “it’s really hard to get communication going”. Clinicians found that they had to adapt their own communication styles to meet the needs of the patient, with Participant 2 describing the need to “speak the same language as her (patient with ASD)”. Participant 6 noted the need for “clear and unambiguous instructions”, and the need to go into more detail in some cases. Participant 9 similarly highlighted that “I am very careful about the things that I say being taken literally so I might not be thinking about using context humour or metaphors…, I would be keeping it very simple”.

Participants also reported that they found that patients with ASD appeared to be “less emotional- like they’re more closed off” (Participant 8). As well as appearing less emotional, patients with ASD found it harder to communicate their emotions to the clinician: “they can often have a really poor ability to understand and identify what they’re thinking and feeling” (Participant 5). Participant 1 suggested that patients with ASD approached emotional identification from a more logical perspective, if at all: “you hit the emotions and then they draw back, they don't want to know. Or they, not necessarily don't want to know, they put a practical take on it. You know, keep the emotions at a level that they then mentally work through it”.

A key problem clinicians raised in treating individuals with comorbid ASD and AN was the difficulty in differentiating between the eating disorder and the ASD, particularly in terms of rigid thinking patterns and routines. Participant 1 noted that the rigidity of ASD made tackling AN symptoms difficult:

“I think that the problem with eating disorders is the rigidity of it- is that patients tend to hate change, hate anything different, very rigid rules around food. And it sounds like an eating disorder but may not be, but just their [individuals with ASD] rigid rules around things. They might eat the same food every day or have three different foods that they eat and they can’t bear to try anything else, they just can’t bear it and that’s quite hard to shift.”

Participant 3 suggested that she would try and judge to what extent the patient’s rigidity was due to their AN and could therefore be improved:

“Because there’s an element of overlap between the ASD diagnosis and the rigidity that comes with an eating disorder, it’s quite useful, at least in my head, if I can start thinking about what, where there could be some flexibility, and where there probably isn’t going to be some flexibility.”

Whereas treatment for EDs would typically focus on changing symptoms, Participant 5 highlighted that treating patients with comorbid ASD often entailed a process of acceptance and adaptation for both the patient and the clinician:

“The other thing is difficulties with rigidity and routine and being realistic about how much flexibility you’re going to achieve with these patients. Helping people to live with the fact- in fact, helping people to accept and be ok with the fact that they have that type, have that approach to plans and routines. And that can be really helpful. And working at level of flexibility which is going to give them some movement in their life without asking too much of them.”

Only one participant mentioned that individuals with comorbid ASD may have additional sensory problems that might complicate the treatment of their ED:

“She was really sensory around food and textures and so thinking about really involving the dietician carefully, and prepping the dietician and co-working with the dietician around what they’re going to suggest. So it kind of helped around those sorts of things… And then I guess just being mindful of some of the other sensory things is really important as well. I had worked with another patient who found sound and other people very activating for her. And just helping her to manage that and accept that, and to explore ways of managing it which is going to be helpful.” (Participant 5).

### Techniques used to treat comorbid ASD/AN

Clinicians noted a number of techniques or approaches that they had previously used in treating patients with comorbid ASD/AN. A common theme was that of a patient-driven process, where the clinician would specifically ask the patient what they found helpful:

“Basically would just ask her along the way, you know like questions checking in with her around, you know, the work we were doing and whether she thought she was getting stuck with ASD routines and habits, or it was more like her eating disorder that was in play. So kind of like trying to tailor it with her ASD in mind.” (Participant 2).

Therapists noted that they would adapt certain aspects of their therapeutic approach:

“And do lots of work around emotional identification and thought identification in session, and at a level that you might do even with young people or teenagers, or younger people and younger children, around that.” (Participant 5).

“I would be keeping it very simple, very basic and be doing a lot of work around mentalising as well in a way that I way if someone has got personality disorder or borderline personality disorder”. (Participant 9).

Individual participants also noted a range of smaller adaptations that they used within treatment, including involving family members in therapy to help with communication (Participants 4, 5, 8), and maintaining a routine with appointment time and location (Participant 9). Participant 8 noted that she had previously used a range of techniques to help with communication and building a relationship with the patient:

“What I do is that I ask them to write like, like write things before session and then to read it or ask me to read it so that it’s easier for them to write it down. I’ve done sessions where they actually don’t look at me. So the chair is turned, and they felt more comfortable talking to alternative objects- so they would bring something like I had a patient with a teddy bear, and she would talk to the teddy bear.”

Similarly, Participant 5 had previously used a number of approaches to aid with emotional identification:

“Lots of work around emotional identification and thought identification in session, and at a level that you might do even with young people or teenagers… around that. Like using faces with different emotional expressions on them and using that as a crib sheet that you have in session all the time, and asking them to point to what they’re feeling on there. I get them to colour in different colours to help them represent it.”

## Discussion

Despite literature suggesting that individuals with both AN and ASD have specific treatment needs, at present there has been no research exploring how clinicians treat this clinically significant common comorbidity, with estimated prevalence rates in AN ranging between 23 and 30% [8, 10]. The findings of this study suggest that clinicians face specific challenges in treating patients with comorbid AN and ASD, and that these challenges that may be compounded by a lack of clinician confidence.

The majority of clinicians interviewed suggested that they lacked confidence or experience in treating individuals with comorbid AN and ASD. Whilst the staff team structure of the eating disorder unit enabled clinicians to consult with more experienced colleagues for support in these cases, it may be possible that staff would benefit from a pathway or guide advising on best practice in these cases. Having a developed pathway could also benefit the referral process for clinicians who have patients with suspected ASD but no official diagnosis: firstly, a pre-arranged referral pathway to specialist services could speed up the diagnostic process. In addition, experienced clinicians noted that they would delay referral until the patient was weight restored to ensure that the apparent ASD traits were not as a result of starvation: it may therefore be appropriate to include a minimum BMI threshold in this diagnostic process to protect against misdiagnosis. This association between ASD traits and traits caused by food restriction in AN recognised by experienced clinicians reflects research suggesting that although the prevalence of ASD is higher in AN, these traits may be exaggerated in some cases by the effects of starvation causing a “pseudo- ASD” which resolves with refeeding and weight restoration [32, 41–43].

Clinicians were able to reflect on how they would adapt their approaches to accommodate ASD traits when treating AN. This reflects a strength of the modern therapeutic approach: clinicians are taught to adapt to the specific needs of the patient through a process of formulation [44]. Nonetheless, previous research suggests that patients with comorbid AN and ASD respond less successfully to treatment than their neurotypical counterparts [29, 37]. Issues with treating individuals with AN and ASD raised by the clinicians in this study suggest that treatment may be hindered due to a number of issues, primarily problems with patient/therapist communication and difficulty identifying underlying thoughts and emotions. This provides support previous research speculating that the socio-communicative and emotional profile of ASD may hinder more traditional therapeutic approaches [31]. NICE guidelines recommend that AN is treated using psychological interventions, such as cognitive analytical therapy (CAT) and cognitive behavioural therapy (CBT). However, research has consistently proven that the success of psychological interventions is influenced by the relationship between the therapist and the patient: it is possible that the socio-communicative difficulties noted by the clinicians in this study hinder this process [45]. Moreover, psychological interventions require the use of metacognition and emotional recognition, which research suggests may be hindered in people with ASD [46, 47].

Previous studies examining treatments for individuals with ASD and psychiatric comorbidities have recommended a range of adaptations to programmes such as CBT, including increasing the number of treatment sessions and introducing materials to aid with emotional identification and communication [39]. In this study, clinicians more experienced in treating individuals with ASD had developed similar techniques to combat these problems, including focused work on emotional identification, the use of materials such as emotional crib sheets, involving family members in treatment, and adapting communicative styles. However, at present the experiences of these clinicians are not shared in specific guidelines or training for other staff members, and sharing of expertise relies on clinical supervision and peer support. With CBT adaptations already existing for ASD and comorbid depression and anxiety, the findings of this present study suggest that therapy for ED also requires the development of specific modifications for an patients with both AN and ASD traits [39, 48].

The strengths of this study lie in its use of well-developed qualitative techniques to evaluate an under-researched topic with clear clinical relevance. The study additionally interviewed participants from a range of clinical backgrounds, giving a range of important perspectives. The use of a semi-structured interview technique allowed the exploration of the topic whilst enabling participants to explore and express their views. However, this study represents a preliminary, qualitative study in the growing area of AN and ASD links that deliberately focused on clinician views and experiences, rather than providing an empirical analysis of the effects of comorbid AN and ASD on treatment. Furthermore, the study took place within a specific London NHS ED Service, and so findings cannot be generalised to all clinicians and services. Further research is needed to empirically evaluate treatment modifications necessary for comorbid AN and ASD, in order to create a standardised, empirically supported treatment approach.

## Conclusions

It is clear that individuals with comorbid AN and ASD represent specific treatment challenges to clinicians, and so require specific treatment adaptations [29]. In this study, treatment modifications appear to be the result of individual therapist experience and knowledge, rather than representing a standardised method. A more standardised approach could incorporate the suggestions for treatment recommended by clinicians in this study, thus providing support to clinicians who lack experience in treating this challenging patient group. This approach could take the form of specific guidelines, treatment pathways, or staff training.

## Additional file

Additional file 1: Interview guide: summary of interview question. (DOCX 13 kb)

## Abbreviations

AN: Anorexia nervosa
ASD: Autism spectrum disorder
BMI: Body mass index
CAT: Cognitive analytical therapy
CBT: Cognitive behavioural therapy
ED: Eating disorder
NICE: National Institute for Health and Clinical Excellence
