# Supplementary material for: Clinicians’ views on working with anorexia nervosa and autism spectrum disorder comorbidity: a qualitative study
Source: BMC Psychiatry. 2017 Aug 10;17:292. doi: 10.1186/s12888-017-1455-3 (PMC5553805; doi:10.1186/s12888-017-1455-3)
Supplement: Additional file 1: — Interview guide: summary of interview question. (DOCX 13 kb) [file 12888_2017_1455_MOESM1_ESM.docx]

**Clinicians’ views on working with anorexia and autism spectrum disorder comorbidity: interview guide**

**Question:**

“What do you do if your patient with an eating disorder has an autism spectrum disorder diagnosis or traits?”

***Interviewer free to ask follow up questions at this point depending on themes raised by the participant. Examples include:***

- ***How do you judge the difference between the symptoms of an eating disorder and autistic traits?***
- ***Do you do anything different with these patients?***
